# Ultrasound imaging of apoptosis: high-resolution non-invasive monitoring of programmed cell death in vitro, in situ and in vivo

**DOI:** 10.1038/sj.bjc.6690724

**Published:** 1999-10

**Authors:** G J Czarnota, M C Kolios, J Abraham, M Portnoy, F P Ottensmeyer, J W Hunt, M D Sherar

**Affiliations:** Ontario Cancer Institute and Department of Medical Biophysics, Faculty of Medicine, University of Toronto, 610 University Avenue, Toronto, M5G 2M9, Canada

**Keywords:** ultrasound imaging, apoptosis, chemotherapy, photodynamic-therapy

## Abstract

A new non-invasive method for monitoring apoptosis has been developed using high frequency (40 MHz) ultrasound imaging. Conventional ultrasound backscatter imaging techniques were used to observe apoptosis occurring in response to anticancer agents in cells in vitro, in tissues ex vivo and in live animals. The mechanism behind this ultrasonic detection was identified experimentally to be the subcellular nuclear changes, condensation followed by fragmentation, that cells undergo during apoptosis. These changes dramatically increase the high frequency ultrasound scattering efficiency of apoptotic cells over normal cells (25- to 50-fold change in intensity). The result is that areas of tissue undergoing apoptosis become much brighter in comparison to surrounding viable tissues. The results provide a framework for the possibility of using high frequency ultrasound imaging in the future to non-invasively monitor the effects of chemotherapeutic agents and other anticancer treatments in experimental animal systems and in patients. © 1999 Cancer Research Campaign


*British Journal of Cancer* (1999) **81**(3), 520–527
© 1999 Cancer Research Campaign
Article no. bjoc.1999.0724

### Ultrasound imaging of apoptosis: high-resolution non-invasive monitoring of programmed cell death in vitro, in situ and in vivo

**GJ Czarnota, MC Kolios*, J Abraham, M Portnoy, FP Ottensmeyer, JW Hunt and MD Sherar**

Ontario Cancer Institute and Department of Medical Biophysics, Faculty of Medicine, University of Toronto, 610 University Avenue, Toronto, Ontario, Canada M5G 2M9

**Summary** A new non-invasive method for monitoring apoptosis has been developed using high frequency (40 MHz) ultrasound imaging. Conventional ultrasound backscatter imaging techniques were used to observe apoptosis occurring in response to anticancer agents in cells in vitro, in tissues ex vivo and in live animals. The mechanism behind this ultrasonic detection was identified experimentally to be the subcellular nuclear changes, condensation followed by fragmentation, that cells undergo during apoptosis. These changes dramatically increase the high frequency ultrasound scattering efficiency of apoptotic cells over normal cells (25- to 50-fold change in intensity). The result is that areas of tissue undergoing apoptosis become much brighter in comparison to surrounding viable tissues. The results provide a framework for the possibility of using high frequency ultrasound imaging in the future to non-invasively monitor the effects of chemotherapeutic agents and other anticancer treatments in experimental animal systems and in patients. © 1999 Cancer Research Campaign

**Keywords**: ultrasound imaging; apoptosis; chemotherapy; photodynamic-therapy

The process of apoptosis has become central to the understanding of the development of normal tissues, the carcinogenic process and the response of tumours to anticancer agents (Berry et al, 1990; Meterissian, 1990; Lowe et al, 1994; Luo et al, 1996; Fisher et al, 1997; Thatte and Dahanikar, 1997). Currently, methods for detecting and monitoring this process are invasive and time-consuming. Biopsied tissue must be stained and imaged using fluorescent optical microscopy, for example (Gaurieli et al, 1992). The result is that spatial maps of apoptosis in tissues cannot easily be produced. In this study, we demonstrate that conventional high-frequency ultrasound imaging techniques (Sherar et al, 1987; Pavlin et al, 1987; Bérubé et al, 1992) are exquisitely sensitive to the apoptotic process (Czarnota et al, 1997*a*; Czarnota et al, this study). Real-time non-invasive measurement of apoptosis in living tissues is now possible. We demonstrate that this imaging method may be used in the laboratory to monitor the responses of cells to chemotherapeutic agents that induce apoptosis (Berry et al 1990; Meterissian, 1990; Lowe et al, 1994; Thatte and Dahanikar, 1997), and to visualize the responses of tissues to anti cancer treatments, such as photodynamic therapy (PDT) (Luo et al, 1996; Webber et al, 1996; Fisher et al, 1997). Specifically, apoptotic cells and tissues exhibit up to a sixfold increase in ultrasound backscatter amplitude in comparison to non-apoptotic counterparts. Additionally, the specific subcellular features that permit the apoptotic phenomenon to be visualized have been identified in this study and are related to the changes in the cellular nuclear material that cells undergo during apoptosis, including chromatin condensation (Allegra et al, 1997) and DNA fragmentation. Our use of ultrasound imaging to detect apoptosis in tissues in response to a specific treatment represents the first evidence of the use of a non-invasive imaging modality to detect programmed cell death both ex vivo and in vivo.

*Received 7 December 1998*
*Revised 8 March 1999*
*Accepted 10 March 1999*

*Correspondence to:* GJ Czarnota

### METHODS

### Cell preparation

All cells were prepared for ultrasound imaging using an acute myeloid leukaemia cell culture system (AML-3), an ideal model for studying apoptosis. For any experimental time point or condition, experiments were completed in quadruplicate. For each experiment, approximately $1 \times 10^9$ cells were grown at 37°C in 300 ml α-minimal-media always from frozen stock samples.

To induce apoptosis, five batches of cells were exposed to cisplatinum at 10 μg ml$^{-1}$. This drug is a DNA intercalater that causes a p53-dependent apoptosis in this cell line (Zamble and Leopard, 1995; Czarnota et al, 1997*a*). Cells were treated with cisplatinum for 0, 6, 12, 24 and 48 h. To confirm that apoptosis was occurring, the 24-h sample was examined using light-microscopy, gel-electrophoresis showing DNA laddering, and trypan blue staining, confirming that approximately 95% of the cells underwent apoptosis at this time point. Cells were washed in phosphate-buffered saline (PBS) and counted to ensure equal numbers of cells, and subsequently pelleted in flat-bottom cryo-tubes at 800 ***g*** on a desktop swinging bucket centrifuge. All pellets were approximately the same size, with a diameter of 1 cm and a height of 1 cm.

To arrest cells in mitosis, effectively enriching the mitotic fraction in the cell population, cells were treated with colchicine at a

*Present address: Department of Mathematics, Physics and Computer Science, Faculty of Engineering and Applied Science, Ryerson Polytechnic University, 350 Victoria Street, Toronto, Ontario, Canada M5B 2K3

concentration of 0.1 μg ml$^{-1}$ for 0, 6, 12, 24 and 48 h. By inhibiting microtubule formation this drug arrests dividing cells at the G2/M checkpoint of the cell cycle, corresponding to metaphase of mitosis (Dustin, 1980). In this cell culture system the maximal enrichment of the mitotic population is an increase to approximately 30%.

To help characterize the effects of the drugs, cytometry was carried out using nuclei from approximately $3 \times 10^5$ cells. Cells were lysed after resuspension in 1 ml isotonic buffer (0.2% Triton X-100 in PBS-citrate, 0.1 mg ml$^{-1}$ RNAase, 0.05 mg ml$^{-1}$ propidium iodide) to release the nuclei. This suspension was strained through a fine-gauze mesh to remove cell debris. Following a 30-min incubation at 4°C in the dark, the samples were analysed on a Becton-Dickenson flow cytometer. A cell cycle analysis program, CellFIT 2.01.2 (SOBR), was used to quantify cells with respect to different cell cycle phases.

For investigations of DNA condensation, effects on ultrasound backscatter, pellets of mitotically enriched cells were taken and gently resuspended in 1 ml of PBS. As controls, samples were treated with only DNAase I (Pharmacia) at concentrations of 5413 U ml$^{-1}$ and 10 826 U ml$^{-1}$ and only Triton X-100 (Sigma) at a concentration of 0.1% (w/v). In order to permeabilize cells and permit DNAase I to enter the cells, samples were exposed to both DNAase I and Triton X-100 at the concentrations given above. Digestions proceeded for 30 min at 37°C and were terminated by adding EDTA to a final concentration of 15 mM. All samples were assessed histologically.

### Light microscopy and analysis

To confirm and investigate the morphology of cells at each experimental condition, ultrasonically imaged and duplicate non-imaged samples were saved for haematoxylin and eosin (H&E) staining by fixing 12 h in 10% (w/v) formalin in buffered saline. These cells were embedded in paraffin and processed as histological sections. No histological differences due to ultrasound imaging were observed. Images of pellet cryosections were obtained to confirm that no differences in packing were present (Czarnota et al, 1997*a*).

Light and fluorescence microscopy was carried out using a Zeiss Axioscope 20 (Carl Zeiss, Germany) coupled to a colour Sony CCD camera. Images were recorded digitally on a IBM PC using the Northern Eclipse Image Analysis Software 1.1 (EMPIX Imaging Inc.).

### Ultrasound imaging and backscatter analysis

All cell samples and animal tissue samples were imaged at room temperature immersed in buffered isotonic saline using a custom built high-frequency ultrasound instrument operating at 40 MHz (Czarnota et al, 1997*a*; Sherar et al, 1987). Living animals were imaged using high-viscosity ultrasound gel (ATL Inc., Reedsville, PA, USA) over areas of skin. The focal depth of the instrument is 9 mm, its axial resolution is 38 μm and its lateral resolution, limited by the ultrasound beam width, is 55 μm. The ultrasound probe was positioned such that the focal zone was the same depth in each imaged specimen. All images were digitally recorded and a physical hard-copy was simultaneously produced.

The ultrasound backscatter amplitude for each time-point was assessed in two manners. In the first method, pixel intensities were transformed to relative ultrasound backscatter amplitudes by multiplying the inverse of the transfer function of the electronics of the ultrasound imaging instrument (Czarnota et al, 1997*a*). This corresponds directly to the degree of ultrasound backscatter amplitude. For each sample, measurements were made using 32 images, each $64 \times 64$ pixels in size, cropped from the focal band of images. In the second method A-scans, the individual line scans which are processed to produce two-dimensional ultrasound images were obtained and assessed. Such A-scans are also independent of the instrument's image processing and amplification processes and their analysis is the subject of a future study. Since both methods provided equivalent results the former was used since it provided information in a visual format, did not require the lengthy collection of radio-frequency data, and could be more readily used with live animal specimens.

### PDT and fluorescence analysis

Male Fisher rats were treated with 12.5 mg kg$^{-1}$ of Photofrin II (QLT, Canada) injected intraperitoneally and were kept in a dark environment for 24 h prior to irradiation. A 5.5 mm craniectomy was then created in each side of the rat's skull, avoiding mechanical stress to the underlying cortex. This area was then treated for 30 s using a red laser light with a wavelength of 632 nm and

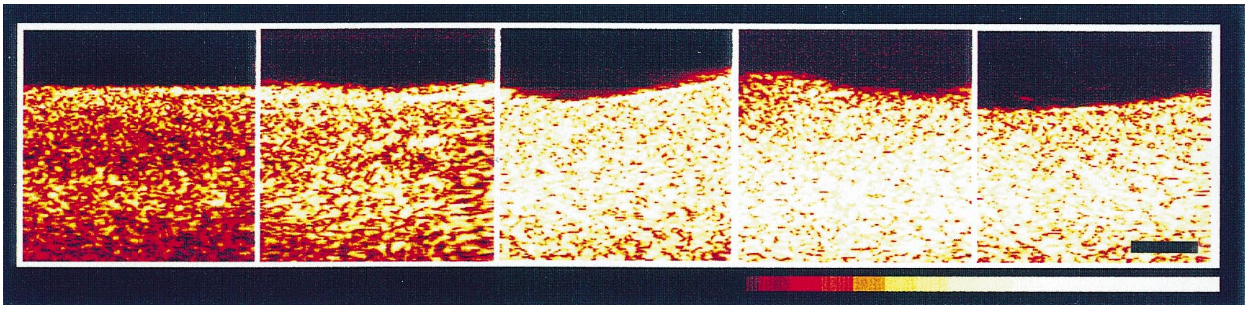

**Figure 1** Results of ultrasound imaging of apoptotic cells. Each panel is a representative ultrasound scan of a pellet of acute myeloid leukaemia cells. The bottom of each ultrasound scan is at the bottom of each frame. Pellets are immersed in buffered saline. From left to right, panels correspond to cells treated with cisplatinum for 0, 6, 12, 24 and 48 h to induce varying degrees of apoptosis. A bar at the bottom right of the figure indicates the colour map used in this image, the left of the bar indicating the colour that corresponds to pixel values of 0 and the right giving the colour that corresponds to a pixel value of 256. At 0, 6, 12, 24 and 48 h histological analysis indicated that 1.6, 2, 36, 87 and 93% of all cells showed nuclear fragmentation, respectively. At the 6-h time point, 72% of the cells exhibited prominent nuclear condensation changing from a nuclear diameter 70% of the cellular diameter before addition of the drug, to a diameter 40% of the cellular diameter at 6 hours. After the 6-h time point, 95% of all cells exhibited nuclear condensation or fragmentation. The speckle pattern is characteristic of ultrasound images. The scale bar indicates 1 mm

© 1999 Cancer Research Campaign

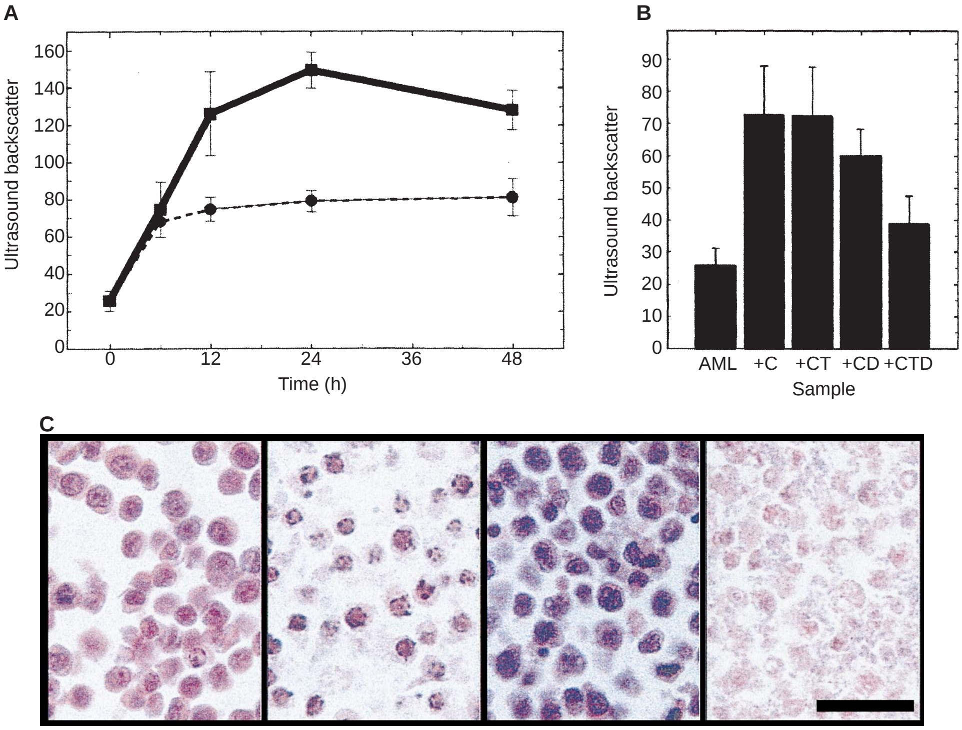

**Figure 2** (**A**) Results of relative ultrasound backscatter measurements for apoptotic and mitotically-enriched acute myeloid leukaemia cells. Relative ultrasound backscatter amplitude is plotted against drug exposure time for cisplatinum-treated apoptotic cells (solid line) and colchicine-treated mitotically enriched cells (dashed line). In the cisplatinum-treated cells, onset of nuclear fragmentation after nuclei have condensed (6 h) further increases the scatter from 2.92-fold to 5.83-fold that of untreated cells. (**B**) Results indicating nuclear condensation is directly associated with increased ultrasound backscatter. Ultrasound backscatter amplitude measurements show that colchicine treated cells (+C) scatter ultrasound 2.83 ± 1.2 times greater than the non-treated cells (acute myeloid leukaemia). Addition of Triton X-100 alone to the colchicine-exposed cells (+CT) did not change the backscatter. Adding DNAase alone to the cells only slightly lowered the backscatter (+CD). Addition of DNAase and permeabilization with Triton X-100 (+CTD) lowered the ultrasound backscatter towards that of the untreated cells. Results shown are for the higher of two DNAase concentrations used. The lower level generated backscatter results intermediate between the backscatter for colchicine exposed cells and samples treated with the higher DNAase concentration. (**C**) Representative histological analyses of acute myeloid leukaemia cells. From left to right panels correspond to untreated viable cells, cells treated with cisplatinum for 24 h (apoptotic), cells treated with colchicine for 24 h (mitotically enriched) and mitotically enriched cells treated with an excess of DNAase I to digest condensed DNA. Control samples treated with DNAase alone or permeabilizing agent alone are histologically equivalent to the mitotically enriched population. The untreated cells exhibited a diameter of 6.0 ± 0.8 μm and had nuclei which are circular in cross-section with a diameter of 4.5 ± 0.8 μm. The cisplatinum-treated apoptotic cells, shown at 24 h after addition of drug, had a slightly larger diameter of 6.4 ± 0.8 μm and prominent fragmented nuclei. On average, each cells had 3.9 ± 2.1 nuclear fragments that were 2.0 ± 1.8 μm in length and 1.3 ± 0.8 μm in width. The colchicine-treated mitotically enriched cells exhibited prominent mitotic figures. These cells were 6.2 ± 1.0 μm in diameter and had nuclear regions 4.3 ± 1.0 μm in mean diameter. After treating such mitotically enriched cells with permeabilizing agent and DNAase, their histological appearance changed (right-most panel). The prominent nuclear staining of the colchicine-treated cells was no longer present and the cells appeared more like untreated cells with respect to their staining characteristics. These cells were unchanged in size after exposure to DNAase; they had a mean diameter of 6.2 ± 1.1 μm and had pink staining nuclear regions that were 4.4 ± 1.3 μm in mean diameter. All values given are ± 1 standard deviation. The scale bar indicates 20 μm a spot size of 3 mm in diameter. This spot size was selected in order to be readily visualized in the 4-mm scan width of the ultrasound microscope next to an untreated region. Several treatment irradiances were employed, including 1, 3, 5 and 17 J cm$^{-2}$. To minimize post-therapy cerebral swelling and still show a sufficient response to therapy, 3 J cm$^{-2}$ was selected for further study. The optical power irradiance at the dural surface was 100 mW cm$^{-2}$. Animal surgery methods that were used are published elsewhere (Jiang et al, 1997).

The animals were sacrificed at three time points: 1.5, 3 and 24 h after the above PDT. The first two time points were chosen to survey early treatment effects. The last time was chosen since earlier experimentation seemed to suggest an accumulation of cells arrested in relatively early stages of apoptosis 8 h post-apoptosis inducing therapy (Li et al, 1996). Equivalent results were obtained whether the rat brains were formalin-fixed prior to ultrasound imaging in order to minimize degradation effects, or imaged ultrasonically prior to fixation.

For general pathological analysis, rat brains were sectioned and haematoxylin and eosin stained. To specifically assess for the effects of apoptosis, an enzymatic method was used (which with terminal-deoxynucleotidyl-transferase) labelled the 3′-OH ends of fragmented DNA with fluorescein-12-dUTP (Promega, Madison, WI, USA) (Gaurieli et al, 1992). Since ultrasound analyses of the 24-h post-therapy specimen were most consistent with the highest levels of putative apoptosis, this specimen was subjected to the apoptosis labelling assay. As a positive control a PDT-untreated rat brain section was treated with proteinase-K and DNAase I, at a concentration of 1 μg ml$^{-1}$ and incubated at room temperature for 10 min prior to staining sections. This method results in about 70–80% positive green-staining cells in the control section, but may stain control cells more intensely that apoptotic cells. As a negative control, contralateral sections of the PDT-treated rat brain were used. Sections of the PDT-exposed rat brain were also stained in this fashion. All sections were counterstained with propidium iodide, which stains both apoptotic and non-apoptotic cells red

 © 1999 Cancer Research Campaign throughout the cytoplasm. Slides of sections were visualized immediately after staining. Microscopy was carried out as above using a standard fluorescein filter set (520 ± 20 nm) and an appropriate filter (> 620 nm) to detect propidium iodide staining respectively. Images of red and green fluorescence were captured separately and combined to form composites. In order to analyse the fluorescence levels within cells in the sections, a computerized approach was used to crop cells after automatic contouring and to quantitatively determine separate levels of red staining and green staining within each cell. These integrated values were then corrected by normalization for slightly different red and green fluorescent background staining values. Image analysis was carried out on IBM PC running Aldus Photostyler Version 2.0.

In rat skin experiments, skin from the dorsal posterior of the animal was shaved. A 1 cm diameter area was exposed to 0, 8.5, or 17 cm$^{-2}$ with an irradiance of 100 mW cm$^{-2}$. Animals were kept in a dark environment before and after treatment. Living animals were imaged in a sedated state (Jiang et al, 1997) 24 h after treatment. Skin biopsies were obtained and submitted for histological analyses. Apoptosis in rat skin was easily visualized in H&E-stained sections. Fluorescently-labelled sections were analysed confirming the presence of apoptosis.

### RESULTS

In order to demonstrate and characterize the efficacy of ultrasound imaging at detecting apoptosis in an ideal setting, a cell-culture system was utilized to permit carefully controlled experiments (Czarnota et al, 1997*a*). Centrifuged cells visualized using a high-frequency ultrasound device (Sherar et al, 1987) operating at 40 MHz and thus permitting higher resolution imaging in comparison to conventional ultrasound devices operating at much lower frequencies, are shown in Figure 1. Individual cells cannot be resolved at the operating wavelength of this instrument but we demonstrate in this study that changes in ensembles of cells can result in significant changes in ultrasound images. The results presented in Figures 1 and 2 indicate that apoptotic cells scatter ultrasound at a level approximately six times that observed with non-apoptotic cells. The degree of scattering also exhibits an approximate linear relation to the progression of apoptosis in terms of nuclear condensation and fragmentation. The ultrasound backscatter amplitude begins to increase as the cells' nuclei condense approximately two- to threefold in diameter over that of nuclei in non-apoptotic cells. The backscatter signal continues to increase to six times that obtained for normal cells with subsequent apoptotic nuclear fragmentation. Representative histological results presented in Figure 2 indicate that these cells undergo a classic apoptotic response. Gross changes in morphology are observable, including changes in the nuclear membrane, nuclear condensation and nuclear fragmentation. We have demonstrated previously that increases in ultrasound image backscatter with apoptosis are due to such differences in cell morphology, and not due to potential differences in cell packing (Czarnota et al, 1997*a*).

Due to the observed correspondence of apoptotic nuclear condensation (Allera et al, 1997) and subsequent nuclear fragmentation with changes in ultrasound images, we hypothesized that the nuclear material in the cells was responsible for the increased ultrasound backscatter. This hypothesis was tested using the same cell culture system, but with the pharmaco-active agent colchicine (Dustin, 1980), which resulted in condensed nuclear material in the form of metaphasic chromosomes, but not fragmentation. Ultrasound results are presented in Figure 2. Whereas the ultrasound images of the apoptotic cells indicated a sixfold increase in backscatter in comparison to normal cells, the mitotic cells exhibited approximately a threefold increase in ultrasound backscatter amplitude. This is in good agreement with the ultrasound levels observed at the 6-h time point after treatment with cisplatinum where the apoptotic fraction of cells exhibits primarily nuclear condensation. By 24 h after colchicine exposure, a maximal cell cycle arrest was visualized histologically and indicated by cytometric analysis to be approximately 30% – the maximal mitotic fraction that the cell line exhibited in response to colchicine.

In order to test whether the presence of condensed DNA is necessary to produce increased ultrasound backscatter, a further series of experiments was devised, the results of which are presented in Figure 2B. These experiments tested the hypothesis that if DNA condensation was responsible for the noted increase in ultrasound backscatter upon exposure to colchicine, then enzymatically removing the DNA condensation (using DNAase) should reduce the ultrasound signal to that of untreated cells. Appropriate controls and histological analyses were included to ensure the drop

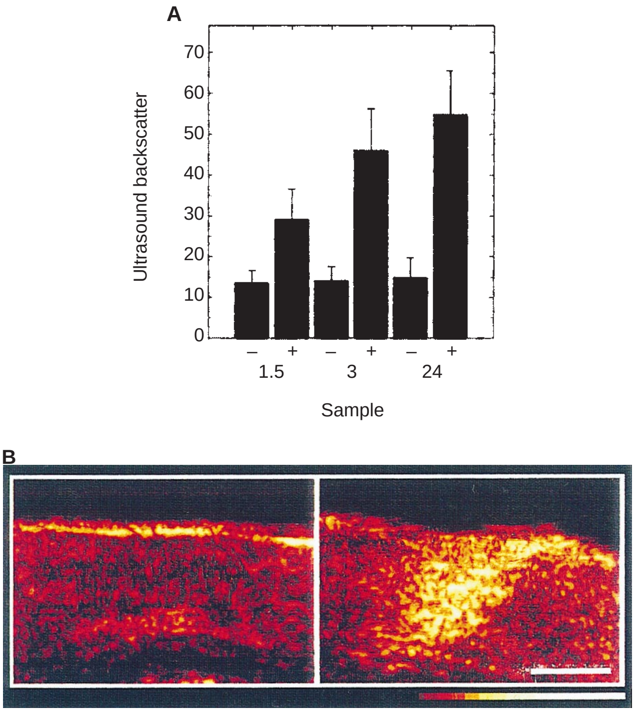

**Figure 3** (**A**) Measurement of ultrasound backscatter amplitude for rat brain treated with photodynamic therapy. Tissue was examined ex vivo 1.5, 3 and 24 h following photodynamic therapy. Results indicate increasing ultrasound backscatter with time compatible with the accumulation of post-photodynamic therapy apoptotic cells. Bars labelled – correspond to non-irradiated controls, whereas bars labelled + correspond to treated samples. Error bars correspond to 1 standard deviation. (**B**) Representative ultrasound image of photodynamic therapy-treated brain tissue examined ex vivo. The left panel corresponds to non-irradiated control tissue contralateral to the treated tissue shown in the right panel. The cone-shaped yellow area of increased ultrasound backscatter corresponds to the treated region. The tissue shown was freshly excised 24 h after therapy and is not fixed. The contrast is equivalent to that obtained with samples fixed for histology. The colour map indicated by the bar below the right panel is the same as for Figure 1. The scale bar indicates 1 mm

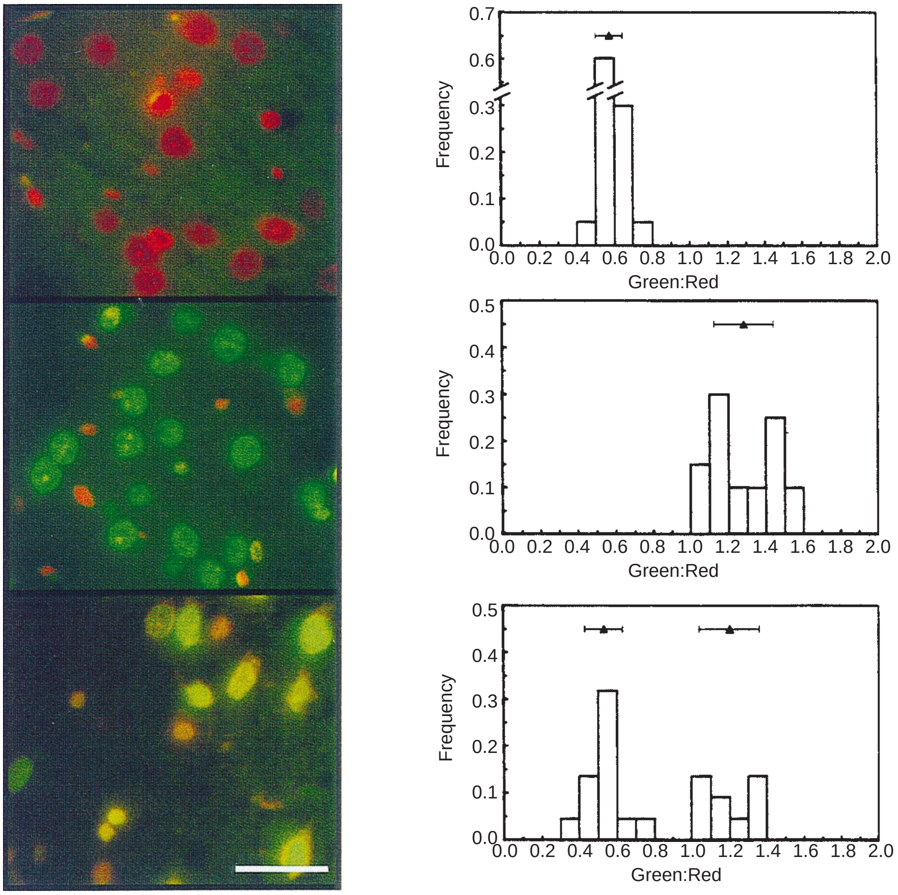

**Figure 4** Representative results of fluorescence microscopy assays for apoptosis. Images in the left column show composite images of propidium iodide and fluorescein fluorescence, whereas right column panels present histograms of the ratio of integrated green staining to integrated red staining for cells cropped from fluorescence images. Images are background normalized. Top panels correspond to data for untreated rat brain – a negative control. Centre panels correspond to a DNAase I-digested rat brain section serving as a positive control for end-stage apoptosis. The bottom panels correspond to the photodynamic therapy-treated brain in the zone of high ultrasound backscatter. Approximately 40% of the cells exhibit green to red staining ratios consistent with the criterion for apoptosis determined using the positive control sample. The level of green staining in the cells increases twofold after photodynamic therapy. The scale bar indicates 20 μm. In the histograms triangles represent mean values and error bars correspond to one standard deviation in ultrasound backscatter signal towards that of the normal cells were a result of the DNAase activity and not the activity of the permeabilizing agent. Using two different concentrations of enzyme, the ultrasound backscatter was found to drop towards a level similar to that of the untreated cells consistent with the interpretation that nuclear condensation is responsible for the increase in backscatter.

To determine whether ultrasound imaging could be used to detect apoptosis that occurred in vivo, we applied the technique to monitor apoptosis ex vivo in an animal system that involved PDT. This type of therapy has been demonstrated to induce apoptosis in several tissues (Luo et al, 1996; Matsumoto et al, 1996; Webber et al, 1996; Fisher et al, 1997; Portnoy et al, manuscript in preparation). In our study, PDT using Photofrin II, a haematoporphyrin derivative, was applied in a rat model system. After an investigation of dose and time effects, tissue responses were monitored at three separate time points. Ultrasound backscatter measurements and ultrasound images obtained at the 24-h time point at the site of therapy and in the non-irradiated contralateral side of an unfixed freshly-excised brain are shown in Figure 3. Backscatter amplitude measurements indicate that the 1.5-, 3- and 24-h post-PDT brain areas exhibited a 2.1 ± 1.1-, 3.3 ± 1.6- and 3.7 ± 2.0-fold increase in ultrasound backscatter, respectively in comparison to individualized controls. The 24-h tissues were selected for histological analysis since ultrasound results indicated a maximal amount of apoptosis in this sample. Since scoring from H&E-stained brain sections was determined to be too subjective in our hands, a specific staining procedure, a TdT assay, was used to detect apoptosis (Gaurieli et al, 1992). Free DNA ends produced by apoptotic chromatin fragmentation were labelled with a green fluorescent stain and a propidium iodide counterstain was used to mark cytoplasm red. A simple computerized assay was utilized where the calculated ratios of green to red staining intensity correlated with the presence or absence of apoptosis. The treated brain section showed two statistically significant populations of cells which were clustered distinctly below and above the apoptotic threshold value of 1 in terms of their green to red staining ratios. Using this value the analysis indicated approximately 40% of the cells to be apoptotic in the treated region (Figure 4). This level of ultrasound backscatter increase corresponded well to cell culture experiments which indicated comparable increases in ultrasound backscatter with apoptosis.

To demonstrate the feasibility of detecting apoptosis in vivo additional experiments were carried out in which a photosensitized

 *© 1999 Cancer Research Campaign*

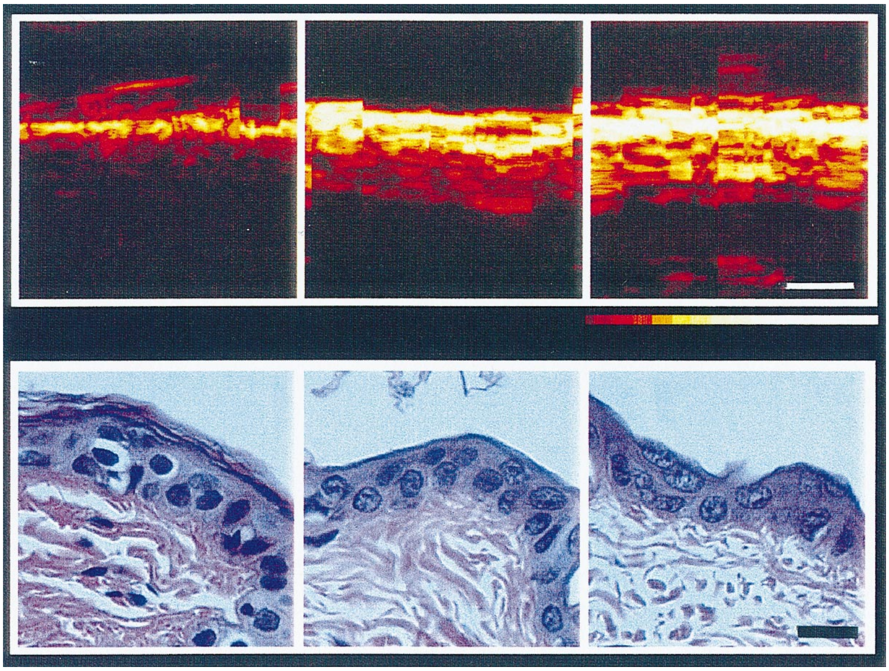

**Figure 5** Imaging of apoptosis in vivo. Top panels present the results of ultrasound imaging, whereas the bottom panels illustrate corresponding representative histological results. From left to right panels correspond to rat skin imaged in vivo 24 h after exposure to 0, 8.5, and 17 J cm$^{-2}$ of activating laser light. Any discontinuities in the images are a result of motions in the living animal caused by its respiration. The colour map shown below the right panel is the same as used in all other figures. The most prominent increase in the top panels occurs at the epidermal surface with increasing dose. The epidermal layer is easily visualized in the left panel – it is the bright line at the top of the skin. An increase in the lower dermal region also occurs. Corresponding histology shows prominent apoptotic cells with condensed and fragmented nuclei in the epidermal region in both the 8.5 and 17 J cm$^{-2}$ samples. A disruption of the cellularity in the dermal region below also occurs with dose. The scale bar in the ultrasound images represents 0.5 mm. The scale bar in the histological images represents 20 μm rat had areas of its skin exposed to activating laser light. Areas of skin in a sedated live animal exposed to 0, 17, and 8.5 J cm$^{-2}$, shown in Figure 5, result in ultrasound backscatter levels that corresponded with the dose of activating light. At doses of 0, 8.5, and 17 J cm$^{-2}$ measurements of ultrasound backscatter amplitude from the epidermis were 12.8 ± 4.3, 32 ± 6.4 and 59.8 ± 17.0 respectively. This part of the skin, which scatters more prominently than the underlying dermis, can be readily identified since the resolution of the instrument axially is 40 μm. Histological analyses revealed increasing levels of apoptosis with the dose of activating light (Figure 5). Consistent with ultrasound levels indicating a marked backscatter response at the epidermal surface, the most prominent levels of apoptosis were exhibited in the superficial cellular layers of the epidermis. Apoptotic cells were easily visible with H&E staining, in contrast to the brain sections, and their identity was confirmed using fluorescent staining for apoptosis. However, increased backscatter was also observed with increasing dose from a less superficial zone consistent with the papillary and reticular layers of the dermis. In this zone the cellular components, fibroblasts and leucocytes, appear to undergo apoptotic cell death with photodynamic therapy resulting in a disruption of the dermal layer. This may account for the increased backscatter seen deep in the tissue, away from the apoptotic cellular epidermis.

### DISCUSSION

This study demonstrates for the first time that apoptosis in tissues is detectable using high-frequency ultrasound imaging. In this investigation, we have been able to demonstrate the ultrasonic detection of apoptosis in specific instances in vitro, ex vivo and in vivo. In the three experimental systems used in this study, acute myeloid leukaemia cells, rat brain and skin tissue, results indicate up to a 25 to 50-fold increase in ultrasound image intensity with the induction of apoptosis.

The in vitro experiments implicate the cell's nuclear chromosomal material as the major source of ultrasound scattering. This is not necessarily surprising since in the chromosome, the fundamental repeating subunit of chromatin, the nucleosome (Czarnota and Ottensmeyer, 1996; Czarnota et al, 1997*b*), is comprised of a protein density of 1.3 g cm$^{-3}$, and a DNA density of 1.7 g cm$^{-3}$ in approximately equal proportions. Some 25 million of the protein units in combination with 2 m of DNA are packed into chromosomes which are approximately 6–8 μm in length when condensed (van Holde, 1988). The process of nuclear condensation, which takes place in the early stages of apoptosis (Allera et al, 1997), similar to mitosis, compacts chromosomes from forms which are distributed throughout most of the cell's nucleus to more canonical condensed forms. Other previous studies also support the postulate that the cellular nuclear material is the major scatterer of high frequency ultrasound (Bérubé et al, 1992; Hunt et al, 1995; Czarnota et al, 1997*a*). Direct evidence providing strong support for this hypothesis is presented in this study, where the induction of DNA condensation and enzymatic degradation of condensed DNA demonstrates such condensation to be sufficient and necessary to obtain an increased ultrasound backscatter. However, in our experiments the mitotically enriched population of cells never exhibited as great an increase in ultrasound backscatter as did the

*© 1999 Cancer Research Campaign* 

apoptotic cells. Specifically, the late-phase apoptotic cells with fragmented nuclei scattered ultrasound approximately twice as much as the early-phase apoptotic cells, with only condensed nuclei. This is consistent with the result that cells in early apoptosis, at 6 h after cisplatinum treatment, also scatter ultrasound less than in later stages in which nuclei are fragmented. A plausible reason for such increases is linked to the randomization of scatterer location (Hunt et al, 1995) within the cell, which would occur with apoptotic nuclear condensation and fragmentation. In preliminary simulations, the condensing of the nucleus, its fragmentation and the spatial randomization of these nuclear components increased the backscatter signal intensity by at least five times. This increase is in agreement with the increases in ultrasound backscatter determined experimentally for the apoptotic cells exhibiting nuclear condensation or nuclear fragmentation.

This study also demonstrates the ultrasonic detection of apoptosis in tissues ex vivo and non-invasively in vivo. In this work, PDT-treated brain tissue was examined ex vivo and the ultrasound imaging technique was used to image skin tissue in vivo in a living animal, treated to induce apoptosis. The role of PDT in inducing apoptosis is currently under investigation. Clearly the effect is toxic to brain tissue, but limited in terms of activation by laser penetration to local regions grossly 8 $mm^3$ in volume. Nevertheless, the ultrasound results in this study coupled with apoptosis-specific fluorescent labelling and analysis of histological sections supports a role for apoptosis in the response to PDT of brain tissue and skin tissue in this experimental system, consistent with other investigations (Luo et al, 1996; Matsumoto et al, 1996; Webber et al, 1996; Fisher et al, 1997; Portnoy et al, manuscript in preparation). In our study the use of ultrasound imaging to detect apoptosis in response to treatment of tissue represents the first evidence of such a modality detecting programmed cell death ex vivo and in vivo induced in a living organism. Since most chemotherapeutic agents are now recognized to induce apoptosis in tumours (Berry et al, 1990; Meterissian, 1990; Lowe et al, 1994; Thatte and Dahanikar, 1997) one may envisage the ultrasound imaging method being used in the future as a rapid means of evaluating the effects of such treatment regimens in vivo. The ultrasound imaging approach demonstrated in this study, once proven in tumour-based models, could potentially provide the clinician with direct and quantitative non-invasive measures of cellular response immediately after chemotherapy, a promising alternative to the current custom of assessing outcome after a complete course of treatment is finished.

This report presents the results of our first study aimed at detecting apoptosis in cells and non-tumour-bearing tissues. This work demonstrates that high-frequency ultrasound imaging can detect apoptosis induced by a variety of manners, including cytotoxic drug and photodynamic methods. The responses of tumours to such therapies may be more complex than those visualized in our idealized model systems and deserve separate study in regards to ultrasound imaging. First, the cell line used in this study represents an ideal situation to measure apoptosis due to the fact that a number of toxic stimuli can induce a well characterized apoptotic response (Czarnota et al, 1997*a*). This cell line was selected because facile growth and experimental manipulation can readily provide adequate bulk quantities of packed cells. The use of such cells was selected as a crude approximation to the packed cells in tissues. We also used a skin system to demonstrate that apoptotic tissue could be detected in a living animal using high-frequency ultrasound imaging. Ultrasound imaging is a relatively straightforward process for the visualization of superficial regions, but clearly an alternative approach would be necessary with subcutaneous tissues. This is necessitated by the high attenuation of epidermal and dermal tissues, which limits ultrasound penetration. Developments occurring in transducer technology should soon permit high-frequency transducers to be mounted on catheters or needles which could aid in the imaging of subcutaneous structures. Lastly, the apoptotic response in tumours to toxic agents and therapies may be more difficult to monitor by ultrasound imaging in comparison to the cell system used and the tissues investigated in our study. Unlike our model systems, chosen deliberately for being relatively homogeneous, tumours are more heterogeneous in terms of consistency. This heterogeneity arises out of angiogenesis, necrosis, infiltration by mononuclear cells and the presence of stroma in addition to viable tumour cells. Treatment of tumours would not only induce apoptosis but cause necrosis and mitotic arrest, all of which could potentially confound ultrasound image interpretation. Such issues would have to be addressed before attempting to use the ultrasound detection of apoptosis in a clinical evaluation. One promising possible manner that may help to distinguish between necrotic, apoptotic and mitotic tissues would be to use spectral analysis on the A-scan information used to form the B-scan images shown in this study (Lizzi et al, 1986). Our preliminary analyses show that this adjunct method is more sensitive to the subtle differences between such cell types (Kolios et al, in preparation).

In conclusion, high-frequency ultrasound imaging was used to detect apoptosis induced by anticancer agents in vitro, and to visualize programmed cell death ex vivo and in vivo. Experimental evidence supports the basis for the ultrasonic detection of programmed cell death to be the subcellular nuclear changes cells undergo during apoptosis. The results indicate that such subcellular structural changes have a profound influence on ultrasound images and provide a framework for future experiments aimed at demonstrating the possibility of rapidly and non-invasively monitoring the effects of chemotherapeutic agents and other anticancer treatments using an ultrasound-based approach.

### ACKNOWLEDGEMENTS

We thank Allan Fernandes, Yew Meng Heng, Arthur Worthington, Kasia Harasiewicz, Karen Hahn and Matthew Skinner for technical assistance. We thank Dr Brent Zanke and Dr Iain Quirt for helpful discussions. This work was supported by research awards to GJC from the Faculty of Medicine, University of Toronto with funds in part from the Medical Research Council of Canada. Additional support was from funds from the MRC awarded to JWH, and FPO, and from funds from the National Cancer Institute of Canada awarded to MDS. MCK is a recipient of a University of Toronto Open Doctoral Fellowship and an Ontario Graduate Fellowship.

### REFERENCES

Allera C et al (1997) The condensation of chromatin in apoptotic thymocytes shows a specific structural change. *J Biol Chem* **272**: 10817–10822

Berry MA, Behnke CA and Eastman A (1990) Activation of programmed cell death (apoptosis) by cisplatin, other anti-cancer drugs, toxins and hyperthermia. *Biochem Pharmacol* **90**: 2353–2362

Bérubé LR, Harasiewicz K and Foster FS (1992) Use of a high frequency ultrasound microscope to image the action of 2-nitroimidazoles in multicellular spheroids. *Br J Cancer* **65**: 633–640

 © 1999 Cancer Research Campaign

Czarnota GJ and Ottensmeyer FP (1996) Structural states of the nucleosome. *J Biol Chem* **271**: 3677–3683
Czarnota GJ, Kolios MC, Vaziri H, Benchimol S, Ottensmeyer FP, Sherar MD and Hunt JW (1997*a*) Ultrasound biomicroscopy of viable, dead and apoptotic cells. *Ultrasound Med & Biol* **23**: 961–965
Czarnota GJ, Bazett-Jones DP, Mendez E, Allfrey VG and Ottensmeyer FP (1997*b*) High resolution microanalysis and three-dimensional nucleosome structure associated with transcribing chromatin. *Micron* **28**: 419–431
Dustin P (1980) Microtubules. *Sci Am* **243**: 66–76
Fisher AMR, Danenberg K, Banerjee D, Bertino JR, Danenberg P and Gomer CJ (1997) Increased photosensitivity in HL60 cells expressing wild-type p53. *Photochem Photobiol* **66**: 265–270.
Gaurieli Y, Sherman Y and Ben-Sasson SA (1992) Identification of programmed cell death in situ via specific labelling of nuclear DNA fragmentation. *J Cell Biol* **119**: 493–501
Hunt JW, Worthington AE and Kerr AT (1995) The subtleties of ultrasound images of an ensemble of cells: simulation from regular and more random distribution of scatterers. *Ultrasound Med Biol* **21**: 329–341
Jiang F, Lilge L, Logie B, Li Y and Chopp M (1997) Photodynamic therapy of 9L gliosarcoma with liposome-delivered photofrin. *Photochem Photobiol* **65**: 701–706
Li YQ, Guo YP, Jay V, Stewart PA and Wong CS (1996) Time course of radiation-induced apoptosis in the adult rat spinal cord. *Radiother Oncol* **39**: 35–42
Lizzi F, Ostromogilsky M, Feleppa E, Rorke M and Yaremko M (1986) Relationship of ultrasonic spectral parameters to features of tissue microstructure. *IEEE Transactions on Ultrasonics, Ferroelectrics, and Frequency Control* **33**: 319–329
Lowe SW, Boris S, McClatchey A, Remington L, Ruley HE and Jacks T (1994) p53 status and the efficacy of cancer chemotherapy. *Science* **266**: 807–810
Luo Y, Chang CK and Kessel D (1996) Rapid induction of apoptosis by photodynamic therapy. *Photochem Photobiol* **63**: 528–534
Matsumoto Y, Muro Y, Banno S, Okasaki M and Tamada Y (1996) Differential apoptotic pattern induced by photodynamic therapy. *Arch Derm Res* **289**: 52–54
Meterissian SH (1990) Apoptosis: its role in the progression of and chemotherapy for carcinoma. *J Am College Surg* **184**: 658–666
Pavlin CJ, Sherar MD and Foster FS (1990) Subsurface ultrasound microscopic imaging of the intact eye. *Ophthamology* **97**: 244–250
Sherar MD, Noss MB and Foster FS (1987) Ultrasound backscatter microscopy images the internal structure of living tumour spheroids. *Nature* **330**: 493–495
Thatte U and Dahanikar S (1997) Apoptosis: clinical relevance and pharmacological manipulation. *Drugs* **54**: 511–532
van Holde KE (1988) *Chromatin.* Springer-Verlag, New York
Webber J, Luo Y, Crihy R, Fromm D and Kessel D (1996) An apoptotic response to photodynamic therapy with endogenous protoporphyrin in vivo. *Photochem Photobiol* **35**: 209–211
Zamble DS and Leopard CJ (1995) Cisplatin and DNA repair in cancer chemotherapy. *Trends Biochem Sci* **20**: 435–439

*© 1999 Cancer Research Campaign* 
